# Effect of Graphene Characteristics on Morphology and Performance of Composite Noble Metal-Reduced Graphene Oxide SERS Substrate

**DOI:** 10.3390/molecules26164775

**Published:** 2021-08-06

**Authors:** Tajana Kostadinova, Nikolaos Politakos, Ana Trajcheva, Jadranka Blazevska-Gilev, Radmila Tomovska

**Affiliations:** 1 POLYMAT, Facultad de Ciencias Químicas, University of the Basque Country UPV/EHU, Joxe Mari Korta Zentroa, Tolosa Etorbidea 72, 20018 Donostia-San Sebastián, Spain; tajana@tmf.ukim.edu.mk (T.K.); mikolaos.politakos@ehu.eus (N.P.); ana.trajcheva@polymat.eu (A.T.); 2 Faculty of Technology and Metallurgy, Ss. Cyril and Methodius University in Skopje, Rudjer Boskovic 16, 1000 Skopje, North Macedonia; 3 Ikerbasque, Basque Foundation for Science, Maria Diaz de Haro 3, 48013 Bilbao, Spain

**Keywords:** graphene aspect ratio, reduced graphene oxide, silver nanoparticles, gold nanoparticles, SERS

## Abstract

Graphene/noble metal substrates for surface enhanced RAMAN scattering (SERS) possess synergistically improved performance, due to the strong chemical enhancement mechanism accounted to graphene and the electromagnetic mechanism raised from the metal nanoparticles. However, only the effect of noble metal nanoparticles characteristics on the SERS performance was studied so far. In attempts to bring a light to the effect of quality of graphene, in this work, two different graphene oxides were selected, slightly oxidized GOS (20%) with low aspect ratio (1000) and highly oxidized (50%) GOG with high aspect ratio (14,000). GO and precursors for noble metal nanoparticles (NP) simultaneous were reduced, resulting in rGO decorated with AgNPs and AuNPs. The graphene characteristics affected the size, shape, and packing of nanoparticles. The oxygen functionalities actuated as nucleation sites for AgNPs, thus GOG was decorated with higher number and smaller size AgNPs than GOS. Oppositely, AuNPs preferred bare graphene surface, thus GOS was covered with smaller size, densely packed nanoparticles, resulting in the best SERS performance. Fluorescein in concentration of 10^−7^ M was detected with enhancement factor of 82 × 10^4^. This work demonstrates that selection of graphene is additional tool toward powerful SERS substrates.

## 1. Introduction

Because of the graphene unique features, such as high electron mobility and high surface area, followed by exceptional mechanical, thermal, and electrical properties, it attracts a huge interest in various research fields, including materials science and engineering. One of the important applications of graphene is its utilization as a substrate for surface-enhanced Raman scattering (SERS) [1] that allows detection of a very low concentration of chemical or biological molecules. Raman signals are inherently weak, especially when the visible light excitation is used and therefore, a small number of scattered photons are used for the detection of the investigated molecules. However, by insertion of the investigated molecules onto the surface of the SERS substrate, a largely increased Raman scattering is induced because of two simultaneous effects. On the one hand, chemical enhancement mechanism, which occurs due to the established molecule-substrate interaction, building a charge transfer complex and facilitating the charge transfer [2,3,4,5,6,7,8]. On the other hand, in case of metal substrate, such as nano-cast gold (Au) or silver (Ag) surface, resonantly enhanced field allows an electromagnetic enhancement effect that much strongly enhances the Raman signal [9,10,11,12,13,14]. Additionally, graphene efficiently quenches the fluorescence resulting in improved quality of the probe molecule spectra [15,16]. As the chemical enhancement in SERS is deemed to arose from the creation of interaction due to vibrational coupling and creation of light-induced charge transfer among the molecule and the substrate, stronger the interaction higher is the chemical enhancement [17,18]. Graphene oxide (GO) and reduced graphene oxide (rGO) are progressing as adequate materials since their ubiquity has also been linked with greater SERS effects. It has been stated that single-layer graphene shows higher SERS signal enhancement in contrast to few-layer graphene [19].

Lately, several experimental attempts were reported concerning the enhancement of the SERS performance by connecting the plasmonic nanoparticles formed of silver and gold with graphene structures [20,21,22,23,24,25,26,27]. Song et al. [28] obtained detection of maximum 200–300 times average value enhanced SERS intensities from Ag nanoparticles on graphene sheet (GS) hybrid substrate compared to pure graphene. Nevertheless, Fan et al. [29] reported 2–3 times enhanced SERS excitations from hybrid nanostructure created of AgNP and GO with respect to neat AgNP. However, the performance of the metal nanoparticles is highly reliant on their dimension, structure, crystallinity, configuration, and formation geometry [30]. The effect of various variables on an achieved enhancement was studied. One of the most investigated was the size of the AgNP and Au nanoparticles (AuNP) and their aggregates [31,32,33], reporting that the average value of SERS enhancement factor is 1.6 times increased when the size distribution is reduced to half [28]. He et al. [34] studied the size of AgNP on the SERS enhancement. They reported that the SERS enhancement factor monotonically increases with augmentation of the particle size and the decrease of the distance between the particles in a dimer. As well, the shape of the AgNP and AuNPs was studied. In case when quasi-cubic AgNP were deposited onto graphene, the SERS enhancement factors were 6.53 times in average greater than that of spherical nanoparticles [28]. Zhang et al. [35] have designed GO-wrapped flower-like silver particles, and reported 3.5 times increased SERS signal based on the structure of the hybrid. An interesting study was conducted for dogbone-shaped gold nanoparticles used as colloidal SERS substrates, and it has been shown that the larger dogbone-shaped gold nanoparticles result in weaker limits of detection [36]. Dilong et al. [37] examined the SERS properties of Au nanoparticles with different sizes and shapes and reported that the Au nanospheres arrangements showed great SERS enhancement linked to their composition due to the presence of numerous SERS hot-spots among neighboring AuNPs generated by the electromagnetic coupling.

The control of substrate surface coverage with noble metal nanoparticles is an important parameter when fabricating active material as a SERS substrate. The proportional relation among the surface coverage and the increment of the intensity of SERS signal was presented [38]. When the coverage is doubled, the enhancement factor is as well nearly doubled [28]. Usually, the ultimate particle coverage decreases as the nanoparticle diameter increases. It has been demonstrated that the final particle coverage is decreased with increasing the particle size resulting in reduced hot-spots in the SERS excitation state and consequently decreasing the signal [25]. The responsibility for the highest SERS signal enhancement is related to the nanoscale gaps within nanoparticle dimers and the probe molecule adsorbed in these so-called “hot-spots” [28]. Ding et al. [39] demonstrated that the high coverage of AgNP onto GO led to enhanced SERS signal. This implies that the enhancement effect occurs due to the great electromagnetic coupling between two neighboring AgNPs with a very small gap, resulting in improved SERS signal. 

It is clear that when hybrid SERS substrates were prepared, made of graphene-based materials and noble metal nanoparticles, the focus was placed on the quality, quantity, and structural characteristics of the nanoparticles, as well as their dispersion over graphene material. However, there are quite different graphene-based materials in size and thickness, level of oxidation, presence of heteroatoms, etc. The quality of graphene nanosheets by means of aspect ratio and presence of sp^3^ C defects within the structure influences importantly the properties, especially the electrical conductivity, the capability of interactions, and mechanical resistance [40]. Therefore, one may expect that the interaction of different aspect ratio with different levels of functionalization of the surface will affect the interaction with metal nanoparticles, interactions with probe molecule, and subsequently, the activity as SERS substrate. This is especially true when simultaneous in situ production of noble metal nanoparticles and GO reduction is performed, which is the subject of the present work. For that aim, the different aspect ratio graphenes with importantly different oxidation level were decorated with Ag and Au nanoparticles and they were investigated as SERS substrate using fluorescein (Fl) as probe molecule. To the best of authors’ knowledge, this is the first study where the effect of the different quality of graphene-based materials over the morphology of the SERS substrates was studied and provides useful information for selection of graphene material for designing a SERS substrate and optimization of their performance. Even though the graphene provides chemical enhancement to the hybrid substrate, which is much lower than the electromagnetic field enhancement from the NPs, the present work demonstrated that by variation of the graphene characteristics, the NPs structuring on the surface is influenced and thus, the SERS performance.

## 2. Materials and Methods

Two types of GO in aqueous dispersion were used (Table 1). The first one, supplied by Graphene Supermarket, (Ronkonkoma, NY, USA), (GOS), has the following characteristics: 60% single layer with concentration of 5 mg/mL, low aspect ratio of 1000 and lower content of oxygen functional groups, and other heteroatom (~20%). The second one was supplied by Graphenea, (San Sebastian, Spain), (GOG) with the following characteristics: about 14,000 aspect ratio, 95% one-layer platelets, with concentration of 4 mg/mL, high amount of oxygen functional groups, and heteroatoms ~50%.

Fluorescein (Fl, C_20_H_12_O_5_) purchased from Fluka, (Fluka, Madrid, Spain), polyvinylpyrrolidone (C_6_H_9_NO)n with average molar mass of 10,000 (PVP), silver nitrate (AgNO_3_) ACS reagent, (Westborough, MA, USA), ≥99.0% and Gold(III) chloride hydrate 99.995% trace metals basis from Sigma Aldrich, (Madrid, Spain), L (+)-ascorbic acid, 99%, (AsA) from ACROS, (Madrid, Spain), were used as received. Milli-Q water was used in all experiments.

**Scheme 1:**
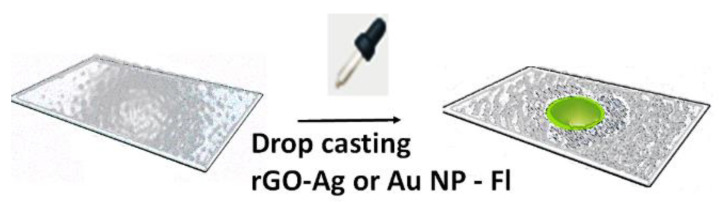
Glass rectangular substrate and sample deposition by drop casting scheme.

For synthesis of the hybrid SERS substrates, first aqueous dispersions were prepared by mixing GO and silver nitrate precursors for Ag nanoparticles (NP) and gold (III) chloride hydrate precursor for AuNP, according to the following procedure. About 50 mL GO aqueous dispersion was sonicated (Hielscher Sonicator-UIS250v, (Hielscher Ultrasonics GmbH, Teltow, Germany) with an amplitude of 70% and energy pulsed at 0.5 Hz at room temperature for 10 min in a 100 mL beaker under continuous agitation of 200 rpm, followed by addition of polyvinylpyrrolidone (PVP) solution (5 mg of PVP in 5 mL water). PVP with molar mass of 10,000 Da was added to assure colloidal stability in the aqueous dispersions during the reduction process. In this dispersion, 60 mL of the respective precursor in aqueous solution was added (60 mg precursor in 2 mL of water). After mixing, the reduction process was performed by ascorbic acid (AsA) reducing agent in concentration of 560 mg in 3 mL water, at room temperature for 72 h. AsA reduced simultaneously the GO and the respective precursor facilitating the growth of the nanoparticles in the dispersions that strongly adsorbed onto the rGO platelets. As a result, hybrid rGO nanoplatelets decorated with AgNP and AuNP were obtained, denoted as: rGOS-AgNP, rGOS-AuNP, rGOG-AgNP, and rGOG-AuNPs according to type of graphene and type of nanoparticles. Total of 4 μL of each of these dispersions was drop casted over glass rectangular substrates and left for drying at standard atmospheric conditions (20 °C and 55% relative humidity, Scheme 1).

The morphology of the composites was investigated by scanning electron microscopy (SEM, (Hitachi TM3030 tabletop model (Krefeld, Germany) at an accelerating voltage of 15 kV. The SEM instrument is equipped with EDX that was used to determine the elemental analysis and mapping of the hybrid substrates. The structure of the GO and distribution of silver and gold nanoparticles decorated onto GO surfaces were investigated with Philips TECNAI G2 20 TWIN transmission electron microscope (FEI, TEM, Barcelona, Spain). For the thermal degradation analysis (TGA) of the materials, a TGA500 apparatus (TA Instruments, Cerdanyola del Valles, Spain) was used. About 2 mg of each material was heated under oxygen ambient from 25 to 700 °C, at a heating rate of 10 °C/min. The SERS activity of the rGO-AgNP and rGO-AuNP hybrid structures was studied by Renishaw Raman Spectrometer (Renishaw, Barcelona, Spain) with an excitation wavelength of 532 nm, 1% of laser power (0.2 mW), 1 s acquisition time, and illumination range from 150 to 3500 cm^−1^. The samples were captured using a 100× long-working distance objective lens and a CCD512 camera.

For SERS performance determination, Fl probe molecules were deposited over each of the hybrid films by drop casting 2 μL of Fl aqueous solution of different concentrations and were left overnight to dry (Scheme 1). Fl spectra onto the rGO/noble metal hybrids were determined up to 10^−7^ M. The blank sample was prepared by drop casting 4 μL Fl aqueous solution with concentration 10^−1^ M onto the neat glass substrate. At least three different spectra were collected for each Fl concentrations. The presented Raman spectra in the manuscript were normalized, using the characteristic peak of the glass substrate onto which the hybrids film were prepared and the Fl was deposited.

## 3. Results and Discussions

The reduction of neat GO in aqueous dispersion was performed with AsA reducing agent in presence of PVP (Mw of 10.000 Da) that sterically stabilized the rGO platelets in dispersions. The simultaneous reduction of GO and respective precursor for formation of Ag and Au nanoparticles resulted in the formation of rGO platelets decorated with AgNP or AuNP. The hybrid nanoplatelets were as well stabilized with PVP.

Elemental composition of all rGO-based materials was determined by EDX and the chemical composition by FTIR spectroscopy. The elemental composition of all materials is presented in Table S1, Supplementary Materials. These results showed that the reduction level is similar in all the samples independently of the presence of Ag or Au precursors, as the relative oxygen content is around 20% in all samples. Carbon content is lower for the composite samples than the neat ones, due to the presence of noble metal nanoparticles on their surface, the content of which is in the range of 7–12%. The exception is rGOG-AuNP material, which presented much lower reduction level and subsequently higher oxygen content and lower AuNP fraction in the composite. It is worth mentioning that the rGOS has similar oxygen content as the GOS (Table 1), which is probably the result of the presence of PVP used for colloidal stabilization of the reduced platelets.

FTIR spectra of the substrates are shown in Figure S1, Supplementary Materials. Both neat rGO materials present typical characteristic peaks of rGO, assigned as follows: ~1710 cm^−1^ (C=O stretching vibration), ~1620 cm^−1^ (C=C graphene domains), ~1400 cm^−1^ (OH deformation vibrations), and ~1030 cm^−1^ (C-O in epoxy). The same peaks appeared in composite substrates made of rGO and Ag or Au nanoparticles, however, certain shifts of each of these vibrations might be noticed with respect to the neat rGO materials, indicating changing of the chemical environment, likely due to the presence of nanoparticles. No important differences in chemical composition can be observed between the different types of rGO and nanoparticles.

**Figure 1:**
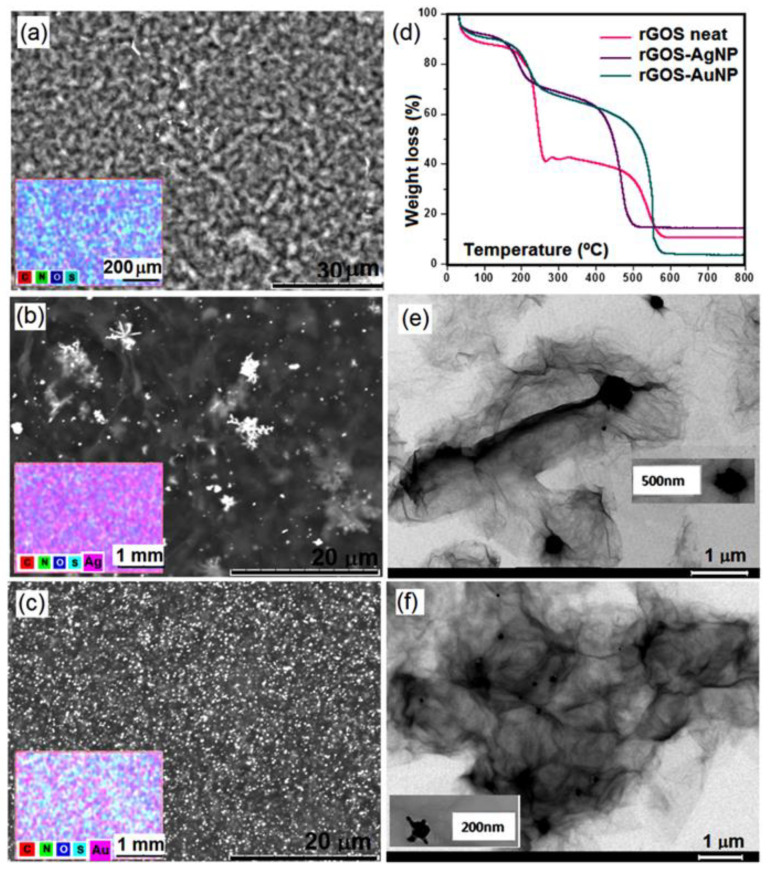
Characteristics of rGOS-AgNP and rGOS-AuNP hybrid films: (**a**) SEM image of neat rGOS, inset: EDX map of the same film; (**b**) SEM image of rGOS-AgNP film, inset: EDX map of the same film; (**c**) SEM image of rGOS-AuNP film, inset: EDX map of the same film; (**d**) TGA curves of hybrid films; (**e**) TEM image of rGOS-AgNP film; (**f**) TEM image of rGOS-AuNP film. In the insets of (**e**,**f**) single Ag and Au nanoparticle is shown, respectively.

In Figure 1 the characteristics of the rGOS hybrid nanoplatelets are shown. rGOS, with aspect ratio of 1000, is characterized with about 20% of oxygen content and about 3% of other heteroatoms, such as sulfur or nitrogen. Figure 1a presents SEM image of the neat rGOS material, in the inset of which, the EDX map is shown, presenting the elemental distribution on the surface of the film. The film is wrinkled and irregular and apparently, the rGOS platelets are covered with PVP, as the map in the inset shows high presence of nitrogen. The morphology of the hybrid rGOS films decorated with AgNP and AuNP are presented in Figure 1b,c, respectively.

Morphology of the hybrid films (Figure 1b,c) differs from that of neat rGOS, as the presence of nanoparticles influenced the drying process and the film quality. AgNP may be observed in Figure 1b (as white structures) in two different morphologies, along with spherical nanoparticles dendrimer-like crystals were observed. The size of the spherical nanoparticles is likely submicron, whereas the dendrimers are large structures of around 10 μm in average. The EDX map presented in the inset of Figure 1b shows that the surface of this hybrid is not covered completely with AgNP, as the graphene structure is still visible (carbon is denoted in red color in the EDX map). The dendrimer silver nanostructures have been observed previously [41], and their formation was explained by a diffusion-limited aggregate model. Namely, in case when growth rate of the nanoparticles is limited by the rate of diffusion of solute atoms to the interface, asymmetric growth of the nanoparticles occurred. Interestingly, such large crystal structures were not observed in case of rGOS-AuNP. As it is shown in Figure 1c, only tiny spherical nanoparticles distributed all over the rGO were obtained. According to the SEM image that represents very good coverage of surface of rGO with AuNP, the EDX–map in the inset of Figure 1c confirms the same, as almost no carbon atoms are visible on the surface of rGO. Even though, Table S1 in Supplementary Materials presents similar Ag and Au NP fractions (9% vs 10%, respectively), these fractions are mass fractions, which means much more moles of Au were presented onto the rGOS. Therefore, the higher quantity of Au nanoparticles along with their much smaller size is the main responsible factors for the higher coverage.

TGA curves of the nanostructures obtained in presence of oxygen are presented in Figure 1d. Except on thermal stability of the composite structures, TGA curves provide information on the chemical composition and interaction between the components. In Figure 1d three weight loss regions are observed. Until 100 °C, the weight loss is assigned to adsorbed water, whereas the weight loss observed between 100 °C and 225 °C corresponds to the oxygen functionalities presented onto the nanostructures. This loss is the largest for the neat rGO, indicating that the hybrid nanoplatelets contain importantly less oxygen functional groups. This means that, either in presence of nanoparticles’ precursors the reduction of GO was more efficient, or the oxygen functionalities were spent in establishing interaction in rGO–nanoparticles, having the second explanation more probable. The degradation of PVP, adsorbed onto nanoplatelets to provide colloidal stability, occurred between 450 and 500 °C. The main loss, corresponding to the graphenic structure, occurred at temperatures higher than 500 °C. The presence of AgNP and AuNP onto the rGOS increased prominently the thermal stability of the hybrid platelets, and the main loss was postponed for about 150 °C, and in the case of AuNP the thermal reinforcement is even more prominent. This indicates that there is higher number of AuNP attached to the rGOS surface, as already shown by EDX (Table S1, Supplementary Materials).

The structure of the hybrids and the size and distribution of the nanoparticles onto the platelets are presented in TEM images (Figure 1e,f). In Figure 1e AgNP aggregates with diameter in a range of 200–500 nm distributed onto the rGO platelets may be observed, as shown in the enlarged image of individual particle in the inset of this figure. According to SEM image of the rGO-AuNP nanostructure (Figure 1f), AuNP are much smaller, therefore better distributed even on the nanoscale level. AuNP formed star-like aggregate with sizes in the range of 20–200 nm, as it is shown in the inset of Figure 1f. The rGO platelets are completely wrinkled and some re-aggregates may be observed (which may be result of the sample preparation).

**Figure 2:**
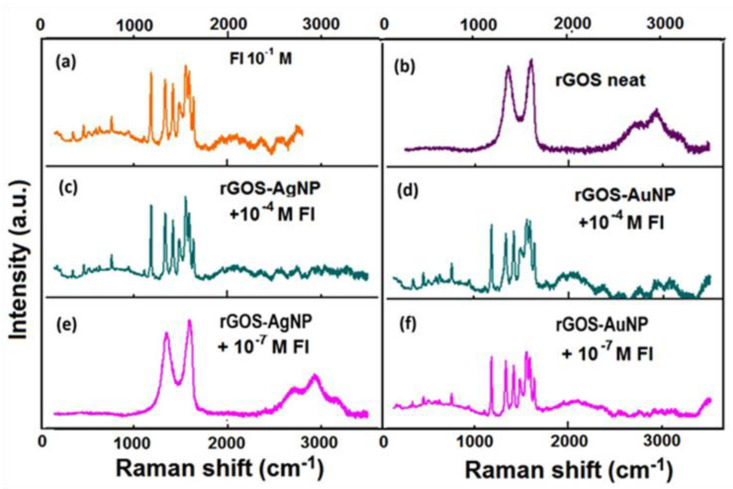
RAMAN spectra of Fl deposited in different concentration over the respective film: (**a**) Fl deposited from 10^−1^ M solution over a glass substrate; (**b**) neat rGOS film; (**c**) Fl deposited from 10^−4^ M solution over a rGOS-AgNP film; (**d**) Fl deposited from 10^−4^ M solution over a rGOS-AuNP film; (**e**) Fl deposited from 10^−7^ M solution over a rGOS-AgNP film; (**f**) Fl deposited from 10^−7^ M solution over a rGOS-AuNP film.

To determine the SERS activity of the prepared films, the Fl probe molecules from aqueous solutions with different concentrations in a range of 10^−1^ to 10^−13^ M were deposited onto the investigated films formed on the glass substrate, followed by characterization by RAMAN spectroscopy. The obtained spectra were compared with these of Fl deposited onto a neat glass substrate and onto the rGOS neat film. The results are presented in Figure 2. It might be observed that the investigated Fl concentration in Figure 2 are up to 10^−7^ M, because bellow this concentration, the Fl was not detected and only characteristic peaks of rGO could be observed, as shown in Figure 2e.

Fl deposited onto the glass substrate (Figure 2a, Scheme 1) presents the typical Fl Raman spectrum with skeletal vibration modes of the xanthene moiety that appears in the frequency range from 1000 to 1800 cm^−1^ [42] Raman spectrum of rGOS is presented in Figure 2b, where the characteristic D band at around 1348 cm^−1^ (originated from the defects in graphenic network) and the G band at around 1600 cm^−1^ (corresponding to the ordered sp^2^ in plane vibration of carbon atoms) [43,44] is observed. The deposited Fl molecules onto the nanostructures containing AgNP onto the surface of rGO presented very nice spectra of Fl, up to Fl concentration of 10^−4^ M (Figure 2c). Further decrease of the Fl concentration deposited onto the nanostructured hybrid films of rGOS-AgNP remains undetectable in the RAMAN spectra, as only the rGO characteristic peaks appeared (Figure 2e). Enhancement factor (EF) was calculated using Equation (1) and the results are presented in Table 2.

(1) $$EF=\frac{I_{SERS}}{I_{RAMAN}}\cdot \frac{{\left[Fl\right]}_{bulk}}{{\left[Fl\right]}_{SERS}}$$

where *I_SERS_* is the intensity of Fl characteristic peak at 1181.8 cm^−1^ in RAMAN spectra measured on the rGOS-AgNP, *I_RAMAN_* is the intensity of the same peak in the RAMAN spectra of neat Fl; *[Fl]_bulk_* is the concentration of Fl deposited on the neat glass substrate and *[Fl]_SERS_* is the concentration of Fl deposited onto the SERS substrate, in this case rGOS-AgNP. 

Table 2 shows that the EF for 10^−4^ M Fl onto rGOS-AgNP is 1373. The determined EF is rather modest and probably is due to the poor coverage of the rGOS with AgNP, large structures and large distance between them (poor coverage), limiting the electromagnetic enhancement effect.

On the other hand, the SERS activity of the hybrid substrate made of AuNPs deposited onto the rGOS nanoplatelets is presented in Figure 2d,f, for Fl concentration of 10^−4^ M and 10^−7^ M, respectively. For both concentrations strong SERS enhancement was obtained, giving rise to EF of 1044 for 10^−4^ M and EF of 82 × 10^4^ for 10^−7^ M concentration of the Fl probe molecule. In this case, the substrate rGOS-AuNP has shown much higher detection capacity than the rGOS-AgNP, probably due to the important difference in the suface morphology and the molar quantity of AuNP. We think that two effects contribute toward this strong enhancement. Namely, as shown in Figure 1c and in the inset, the surface of the rGOS-AuNP film is almost completely covered by the nanosize AuNP. Such covarage ensure smaller distance between the nanoparticles, so called nanoscaled gaps [29] on the rGOS-AuNP than on rGOS-AgNP. Because of that, the former provides enhanced electomagnetic coupling effect between the neighbouring AuNPs [44] and consequently, the electromagnetic enhancement is better than for Ag-containing substrate. Nevertheless, as the TGA results suggested, likely strong interaction rGOS–AuNP were established, based on the important drop of the oxygen functional groups amount. The strong interaction decreased the distance substrate–probe molecule due to the vibrational coupling that creates a light-induced charge transfer complex [17,18], contributing to increase the chemical enhancement. Additional support was obtained by the star-like structure of the AuNP aggregates, as observed in TEM image (Figure 1f), as previously it was demonstrated that non-spherical AgNP positively affected the SERS effect [29]. 

**Figure 3:**
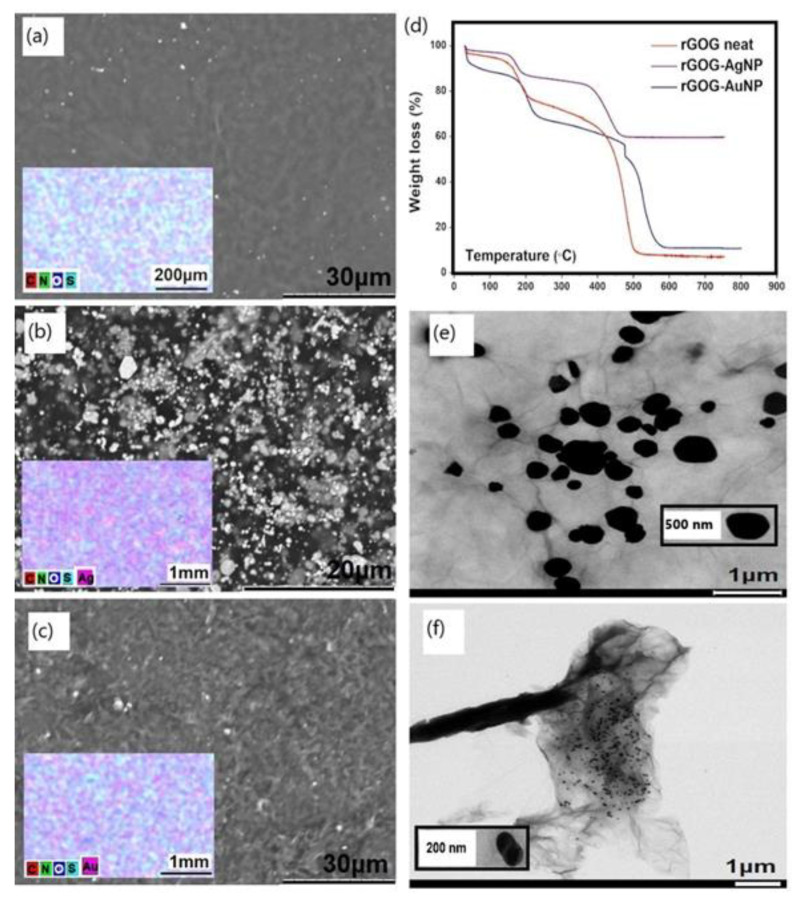
Characteristics of rGOG-AgNP and rGOG-AuNP hybrid films: (**a**) SEM image of neat rGOG, inset: EDX map of the same film; (**b**) SEM image of rGOG-AgNP film, inset: EDX map of the same film; (**c**) SEM image of rGOG-AuNP film, inset: EDX map of the same film; (**d**) TGA curves of hybrid films; (**e**) TEM image of rGOG-AgNP film; (**f**) TEM image of rGOG-AuNP film.

In Figure 3a, the morphology of the neat rGOG sample is shown. According to the EDX map presented in the inset of Figure 2a, the rGOG surface is completely covered by PVP. The morphology of the composite samples rGOG-AgNP and rGOG-AuNP, displayed in Figure 3b,c, respectively, reveals non-regular spherical nanoparticles dispersed onto the surface of rGOG. The maps in the insets of both figures show uniform distribution of the nanoparticles throughout the whole rGOG sheets, however, much better for AgNP. If compared with the respective rGOS SEM images (Figure 1b,c), much more AgNPs with smaller size are distributed onto GOG-reduced platelets. This effect is probably a result of the increased oxidation of the initial GOG (50%) that established more interactions with the Ag+ ions during the in situ formation of the nanocomposite sample than GOS (20%). However, this effect is opposite in case of AuNPs. This can be because Au^3+^ ions are less prone to chemical interactions than Ag^+^ ions, therefore the presence of the multiple functional oxygen groups on the GOG surface has rather negative effect on the AuNP–rGOG interactions. Consequently, the mechanism of coupling the rGOS and nanoparticles is distinct for Ag and Au. In case of Ag, the silver ions adsorbed on the GO surface and the nucleation of the nanoparticles occurred at the surface, whereas in case of Ag, the nanoparticles nucleated in the dispersion and are afterwards adsorbed on the rGOS surface. These results indicate that in case of AuNPs, more naked or less functionalized graphene surface is favorable for the synthesis of composite with well-distributed AuNP.

It is worth noting that the quality of graphene affects not only the distribution and aggregation of the nanoparticles formed on its surface, but also their morphology, probably due to the simultaneous reduction process of both graphene and the precursors. TEM images in Figure 3e present that AgNP forms large aggregates (200–800 nm) with a large distance between them, which means that the possibility for electromagnetic coupling between neighboring nanoparticles is rather low. Figure 1f reveals decreased particle size of AuNPs, which are packed closely onto some of the areas of the rGOG nanoplatelets. Finally, according to TGA results shown in Figure 3d, both nanocomposites presented important difference in the weight loss curves, which demonstrates presence of structural and compositional difference. The thermal degradation behavior is similar to the rGOS-based composites, thus three clear weight loss regions are observed. However, rGOG-AuNP shows similar behavior as the neat rGOG, indicating that the interaction between the AuNPs with the rGOG is not strong. There is very low amount of residuals after the TGA analysis, indicating low amount of AuNPs (according to Table S1, it is 7.3%), not strongly attached to rGOG. In case of rGOG-AgNP nanocomposite, significantly less oxygen functionalities were lost, accounting for better interaction of AgNP with rGOG with respect to AuNP and with respect to rGOS composites. The fact that rGOG is characterized by higher aspect ratios and lower thickness than rGOS means that at the same quantity, rGOG offers larger surface area, thus, lower coverage with nanoparticles is likely achieved than in case of rGOS. However, a very high amount of residual (~60%) indicates that AgNP were very strongly attached to the rGOG.

**Figure 4:**
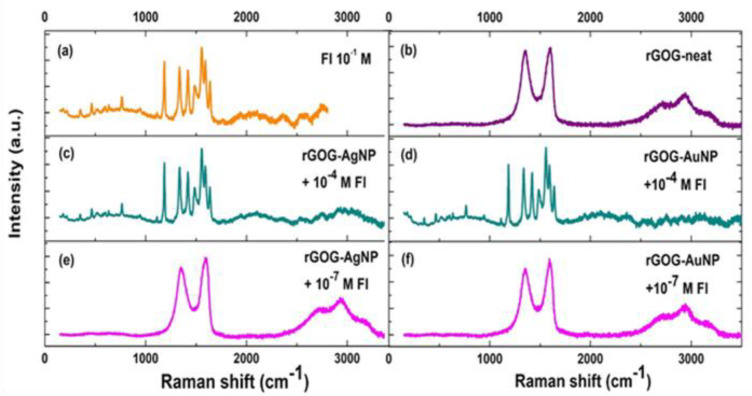
Raman spectra of Fl deposited in different concentration over the respective film: (**a**) Fl deposited from 10^−1^ M solution over a glass substrate; (**b**) neat rGOG film; (**c**) Fl deposited from 10^−4^ M solution over a rGOG-AgNP film; (**d**) Fl deposited from 10^−4^ M solution over a rGOG-AuNP film; (**e**) Fl deposited from 10^−7^ M solution over a rGOG-AgNP film; (**f**) Fl deposited from 10^−7^ M solution over a rGOG-AuNP film.

Figure 4 depicts the Raman spectra of the rGOG-based nanocomposite samples, compared with Fl deposited onto glass substrate and neat rGOG. 

Comparing all the RAMAN spectra presented in Figure 4, it is clear that both nanocomposites rGOG-AgNP and rGOG-AuNP were able to detect Fl in as low molar concentration as 10^−4^. The Fl spectra determined onto these nanocomposites substrates are clear, having all the characteristic peaks with higher intensity than the same in the Fl deposited onto glass substrate in a concentration of 0.1 M. The resulting EFs are 908 for rGOG-AgNP and 1892 for rGOG_AuNP (Table 2). Despite this strong enhancement, both nanocomposite substrates were not able to determine the 10^−7^ M concentration. The EF of rGOG-AgNP is similar to that of rGOS-AgNP; the result can be justified with the morphology and the composition of both samples. Namely, the AgNP formed onto rGOS surface are dendrimer-like structures with tens of microns dimensions, whereas on rGOG surface irregular spherical particles were formed with size in a range of 200–800 nm. Even though the last morphology is likely more prosperous for SERS activity, the large distance between the nanosized structures limits the detection to 10^−4^ M concentration, due to less possibility to establish the electromagnetic coupling. The rGOG-AuNP as well presented limited enhancement until 10^−4^ M Fl concentration. Moreover, its performance as SERS substrate is worse than rGOS-AuNP. Likely, it is due to the less established interaction between both rGOG and AuNP, and, because of the worst coverage of the surface of rGOG. The first effect accounts for the drop in chemical mechanism of enhancement, whereas the second one for the drop in electromagnetic mechanism.

## 4. Conclusions

The simultaneous reduction of GO in the presence of silver nitrate and gold (III) chloride hydrate resulted in successful modification on the surface of rGO with nanoparticles of silver and gold. Two types of colloidal GO were studied, one characterized with small aspect ratio (1000) and lower oxidation level (GOS, 20%) and the second one with much larger aspect ratio (14,000) and densely oxidized (GOG, 50%). The effect of these characteristics over the properties of the nanocomposites, was studied and related to their performance as SERS substrates. It was found that the characteristics of the GO affect importantly the properties and performance of the nanocomposites. 

In case of AgNP onto lower aspect ratio GOS, the nanoparticles developed on the surface of its reduced form are with dendrimer-like morphology, few microns in size, and are less densely packed. Obviously, the nanoparticle nucleation points were the oxygen functionality. Because of that, in case of high aspect ratio and heavily functionalized GOG, the AgNP were smaller but more densely packed. Both of them presented very similar enhancement factor of about 1000 for detection of Fl molecules in concentration of 10^−4^ M. The similar behavior was attributed to the compensated effect of size and shape of AgNP, the distance between them, and the strong interactions. The aspect ratio obviously affected the coverage.

In case of AuNP, the effect of the quality and characteristics of GO were exactly opposite. Besides the light oxidation of GOS, the star-like aggregates of AuNP, developed onto rGOS, were more densely packed, than in case of GOG. As a consequence, the SERS performance of the smaller aspect ratio substrates based on GOS and AuNP presented the best performance for Fl detection, up to 10^−7^ M concentration with very high EF of 82 × 10^4^. The limited enhancement effect of GOG-based substrate up to 10^−4^ M Fl concentration was attributed to lower coverage with AuNP and higher distance between them. 

These results, for the first time to the best of the authors’ knowledge, provide fundamental knowledge about the effect of the quality of graphene on the characteristics of in situ created hybrids with Ag and Au nanoparticles and their performance as SERS substrates.

## Figures and Tables

**Table 1:** Properties and elemental analysis of GOG and GOS.

Properties	GOG	GOS
Concentration	4 mg/mL	5 mg/mL
Elemental Composition	Carbon: 49–56% Oxygen: 41–50% Hydrogen: 0–1% Nitrogen: 0–1% Sulfur: 2–4% (Up to 5% heteroatoms)	Carbon 79% Oxygen 20% (Up to 1% heteroatoms)
Lateral dimension	<10 μm	0.3–0.7 μm
Thickness	Monolayer content (measured in 0.05 wt%): >95%	1 atomic layer-at least 80%
Color	Yellow-brown	Brown
Aspect ratio (lateral dimension/diameter)	~14.000	~1000

**Table 2:** EF values of the hybrid films made of rGOS combination with AgNPs and AuNPs.

Sample	rGOS-Ag	rGOS-Au	rGOS-Au	rGOG-Ag	rGOG-Au
[Fl]	10^−4^ M	10^−4^ M	10^−7^ M	10^−4^ M	10^−4^ M
EF	1373	1044	82 × 10^−4^	908	1892

## Data Availability

All data are already included in the main manuscript and in the Supporting information file.

## Supplementary Materials

The following are available online. Table S1: Elemental analysis of all SERS substrate obtained by EDX. Figure S1: FTIR spectra of the neat rGO materials and their composites with Ag and Au nanoparticles.
