# Supplementary material for: Effect of Graphene Characteristics on Morphology and Performance of Composite Noble Metal-Reduced Graphene Oxide SERS Substrate
Source: Molecules. 2021 Aug 6;26(16):4775. doi: 10.3390/molecules26164775 (PMC8401241; doi:10.3390/molecules26164775)
Supplement: Supplementary file 1 [file molecules-26-04775-s001.zip › molecules-1317993-SI.pdf]

Supporting Information

Effect of Graphene Characteristics on Morphology and Performance of Composite Noble Metal-Based SERS Substrate

Tajana Kostadinova [1], Nikolaos Politakos [2], Ana Trajcheva [3], Jadranka Blazevska-Gilev [4,*] and Radmila Tomovska [5,*]

Table S1. Elemental analysis of all SERS substrate obtained by EDX.

Samples	C p/eV-%	O p/eV-%	N p/eV -%	S p/eV -%	Ag p/eV -%	Au p/eV -%
**rGOS-neat**	75%	22%	1%	2%	-	-
**rGOS-Ag**	68%	20%	1%	2%	9%	-
**rGOS-Au**	64%	23%	1%	2%	-	10%
**rGOG-neat**	77%	19%	1.4%	2.6%	-	-
**rGOG-Ag**	64%	20%	1%	3%	12%	-
**rGOG-Au**	48%	41%	1.2%	2.5%	-	7.3%

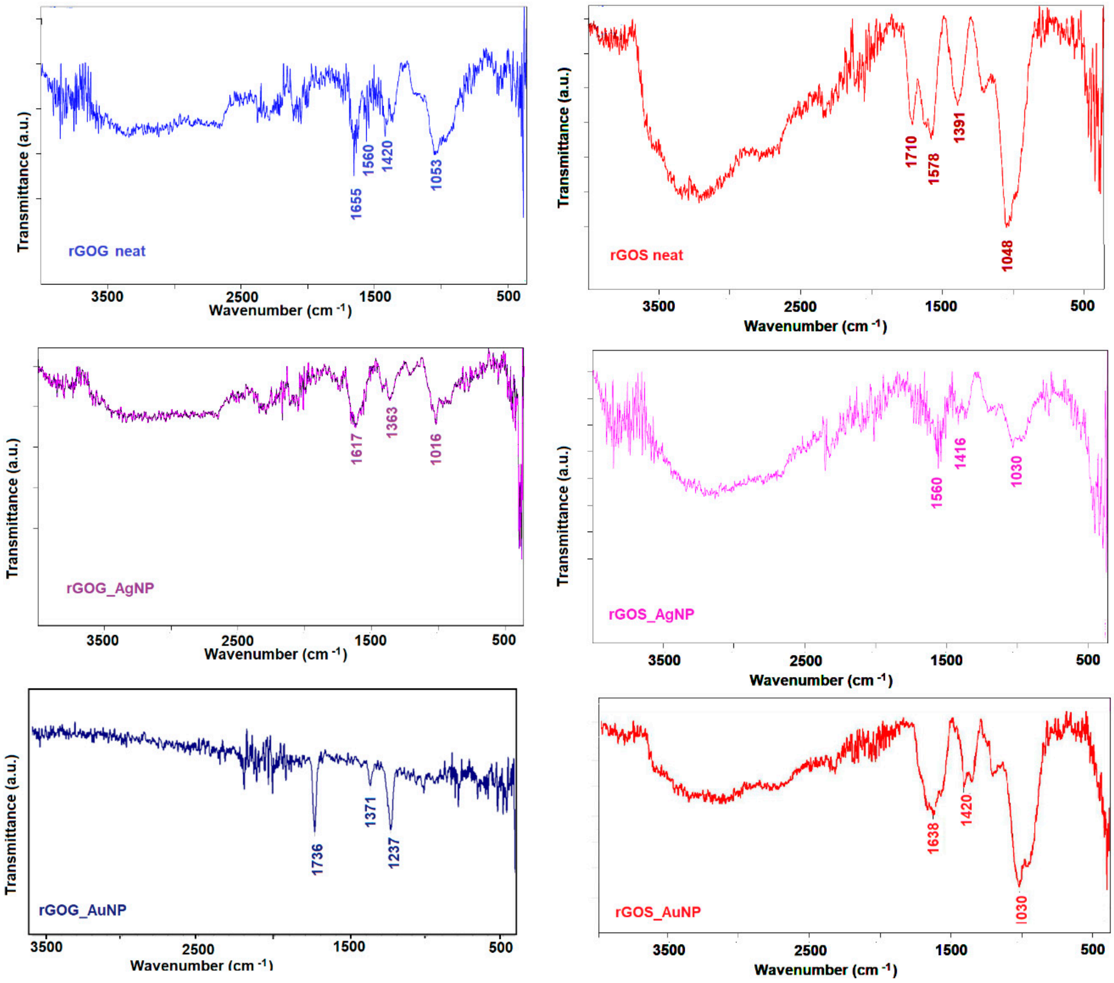

Figure S1. FTIR spectra of the neat rGO materials and their composites with Ag and Au nanoparticles.
